# Intracranial Plasmacytoma Mimicking a Cavernous Sinus Meningioma

**DOI:** 10.7759/cureus.12716

**Published:** 2021-01-15

**Authors:** Stephen J Bordes, Edinson Najera, Michal Obrzut, Hamid Borghei-Razavi, Badih Adada

**Affiliations:** 1 Anatomical Sciences, St. George’s University School of Medicine, St. George’s, GRD; 2 Anatomical Sciences, Tulane University School of Medicine, New Orleans, USA; 3 Neurosurgery, Cleveland Clinic Florida, Weston, USA; 4 Neurosurgery, Neurological Institute, Cleveland Clinic - Taussig Cancer Center, Cleveland, USA

**Keywords:** plasmacytoma, cavernous sinus, radiotherapy, craniotomy, neurosurgery, neurointerventional radiology

## Abstract

Extramedullary plasmacytomas involving the cavernous sinus are rare manifestations of multiple myeloma, and management strategies for such a pathology are not extensively discussed in the literature. In this case report, we describe the case of a patient presenting with a cavernous sinus syndrome secondary to a presumed meningioma. Surgical intervention was avoided as a computed tomography-guided biopsy was performed yielding the diagnosis of a cavernous sinus plasmacytoma. Neurointerventional radiology obtained the cavernous sinus mass biopsy using an approach through the maxillary bone and sinus. Histopathology identified sheets of atypical plasma cells, and the patient was referred to radiation oncology for further management.

## Introduction

Extramedullary plasmacytomas, especially those manifesting in the form of cavernous sinus syndrome, are extremely rare and not well documented in the current literature [1-3]. Extramedullary plasmacytomas are plasma cell tumors presenting outside of the bone marrow. These tumors can occur in the upper airways as lymphocytes and plasma cells are heavily populated in these areas [2]. Cavernous sinus syndrome, a sequela characterized by orbital pain, diplopia, exophthalmos, and ptosis, can be caused by a number of factors, including infection, neoplasm, inflammation, trauma, and vascular etiology [1,2,4,5]. Plasmacytoma is an unusual cause of cavernous sinus syndrome which is more often caused by tumors such as meningiomas, pituitary adenomas and squamous cell carcinomas, aneurysms of the carotid artery, and cavernous sinus thrombosis. Our patient was diagnosed with multiple myeloma six months prior to the manifestation of cavernous sinus syndrome but had also been treated for squamous cell carcinoma of the leg in previous years. A computed tomography (CT)-guided biopsy successfully identified the intracranial lesion and ultimately changed the patient’s management strategy, avoiding a major cranial resection.

## Case presentation

**Figure 1:**
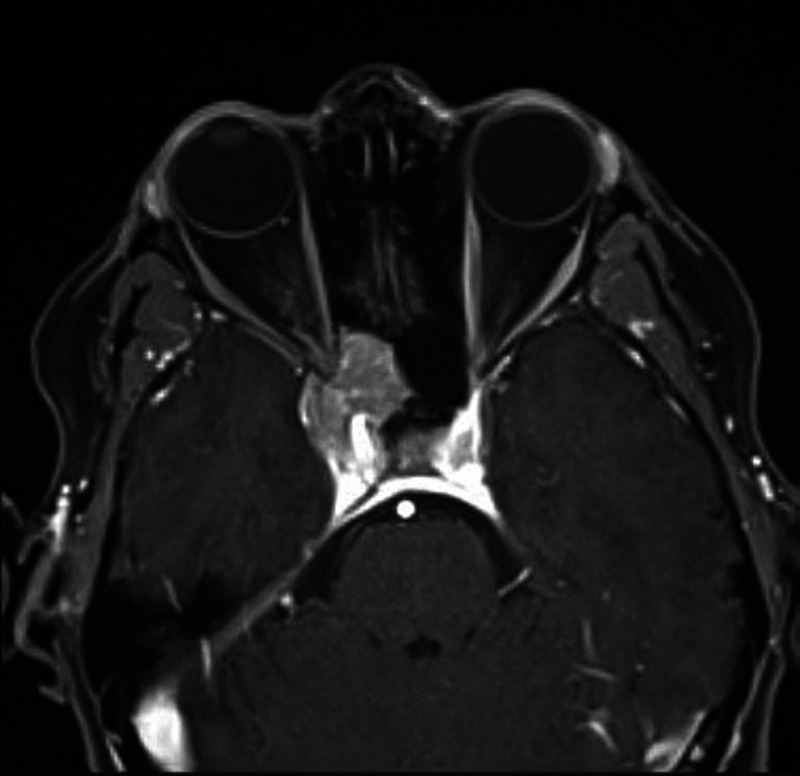
Axial post-contrast T1-weighted MRI showing an enhancing lesion in the right cavernous sinus, later pathologically proven to represent a plasmacytoma. T1, longitudinal relaxation time; MRI, magnetic resonance imaging

**Figure 2:**
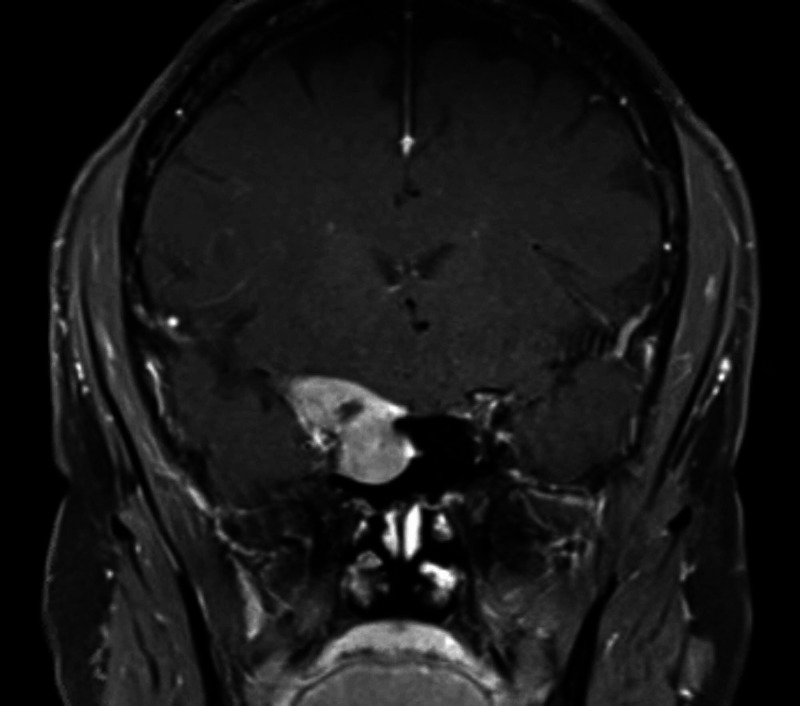
Coronal post-contrast T1-weighted MRI demonstrating intracranial and right sphenoid sinus extension of the plasmacytoma. T1, longitudinal relaxation time; MRI, magnetic resonance imaging

A 61-year-old woman, with a history of squamous cell carcinoma of the lower extremity status post resection, stage IV renal disease secondary to lambda light chain multiple myeloma (diagnosed in February 2020), and poor initial response to systemic chemotherapy, presented to her primary care physician in August 2020 due to headaches and visual auras in her right eye. Within a week, the patient lost all vision in her right eye as well as extraocular muscle movement. Magnetic resonance imaging (MRI) of the brain showed a 2.3-cm right parasellar mass and the patient was referred to neurosurgery with a presumed diagnosis of a meningioma. A repeat MRI the following week showed a 4 × 3-cm extra-axial mass involving the right cavernous sinus with extension to the optic canal, frontotemporal base, and posterior ethmoid and sphenoid sinuses (Figures 1, 2). The mass encased the cavernous right internal carotid artery abutting the right superior rectus muscle. Physical examination was significant for right extraocular muscle plegia, dilated and non-reactive right pupil, right afferent pupillary defect, right periorbital paresthesia, and cranial nerve II to VI palsy. The patient was diagnosed with cavernous sinus syndrome secondary to meningioma versus malignancy.

**Figure 3:**
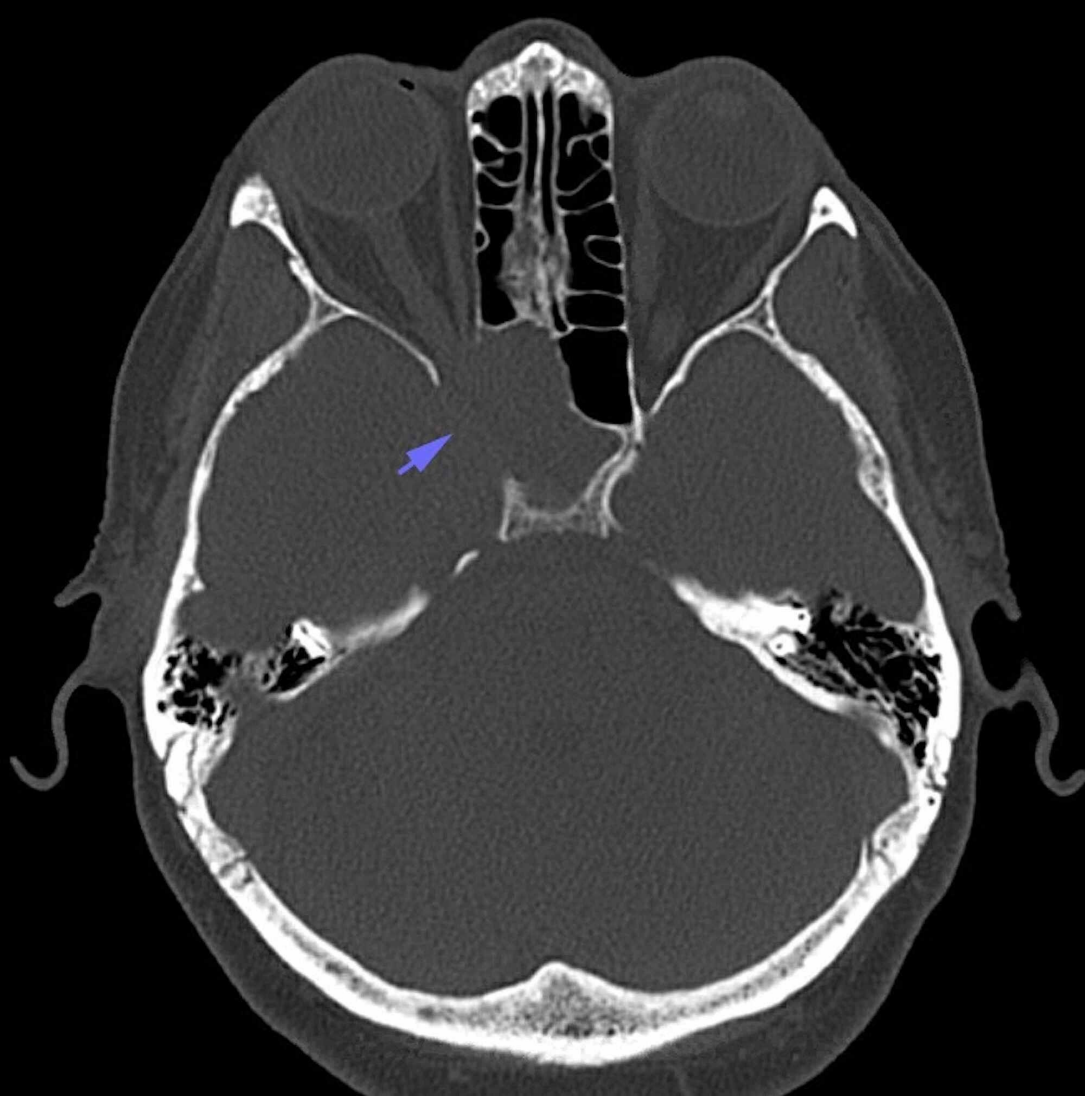
Axial brain CT showing osseous erosion and remodeling by a right-sided plasmacytoma (arrow). CT, computed tomography

**Figure 4:**
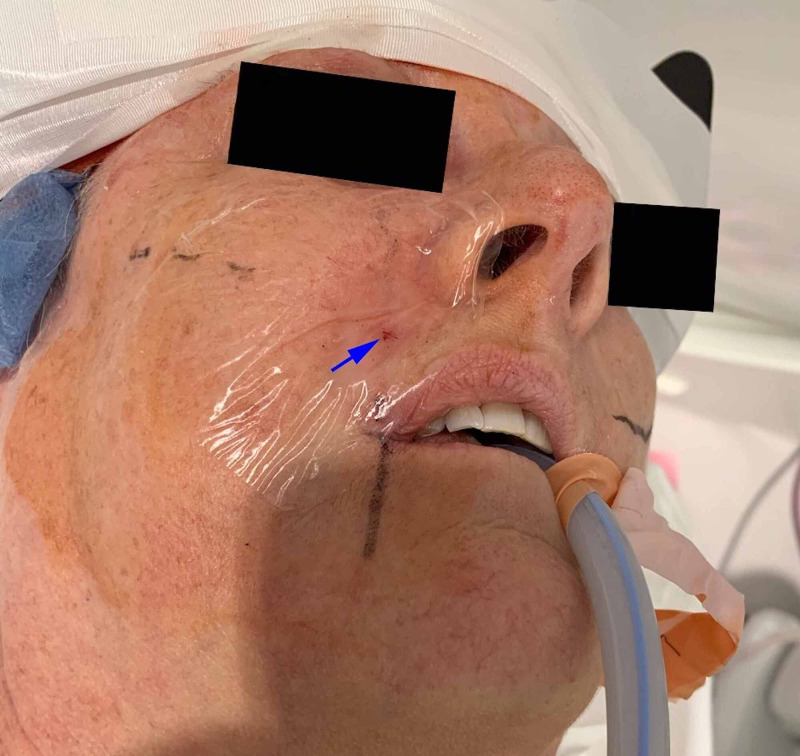
The post-procedure needle access site (arrow) to the right maxillary sinus.

**Figure 5:**
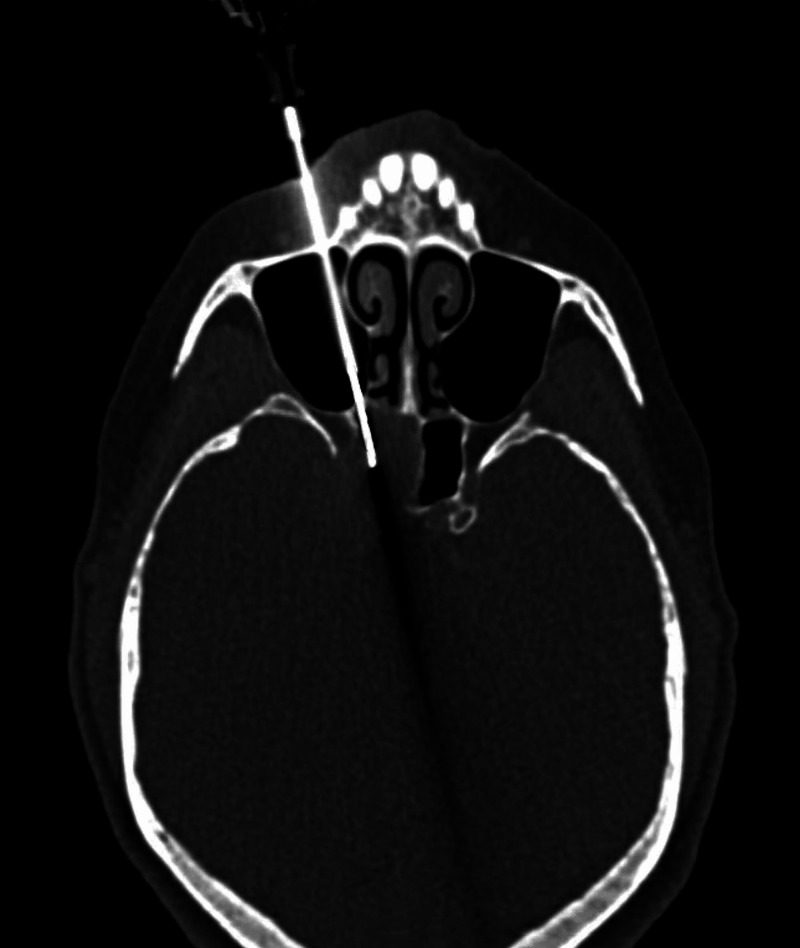
Axial, intraoperative, brain CT showing the path of the guiding needle through the right maxillary sinus and the right sphenoid sinus to the right cavernous sinus plasmacytoma. CT, computed tomography

**Figure 6:**
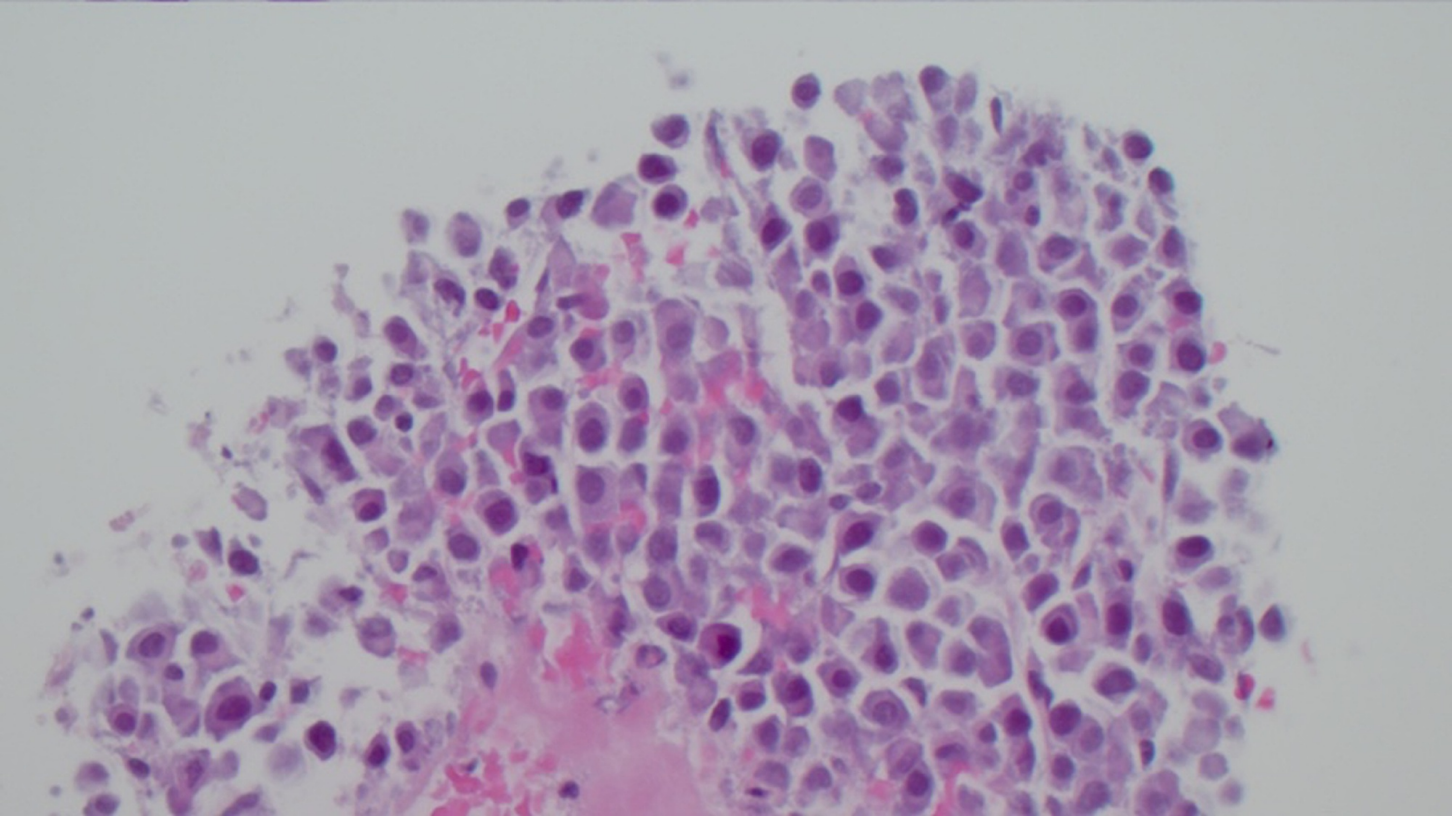
High-power view showing the discohesive malignant plasma cells (H&E, 400×). H&E, hematoxylin and eosin

**Figure 7:**
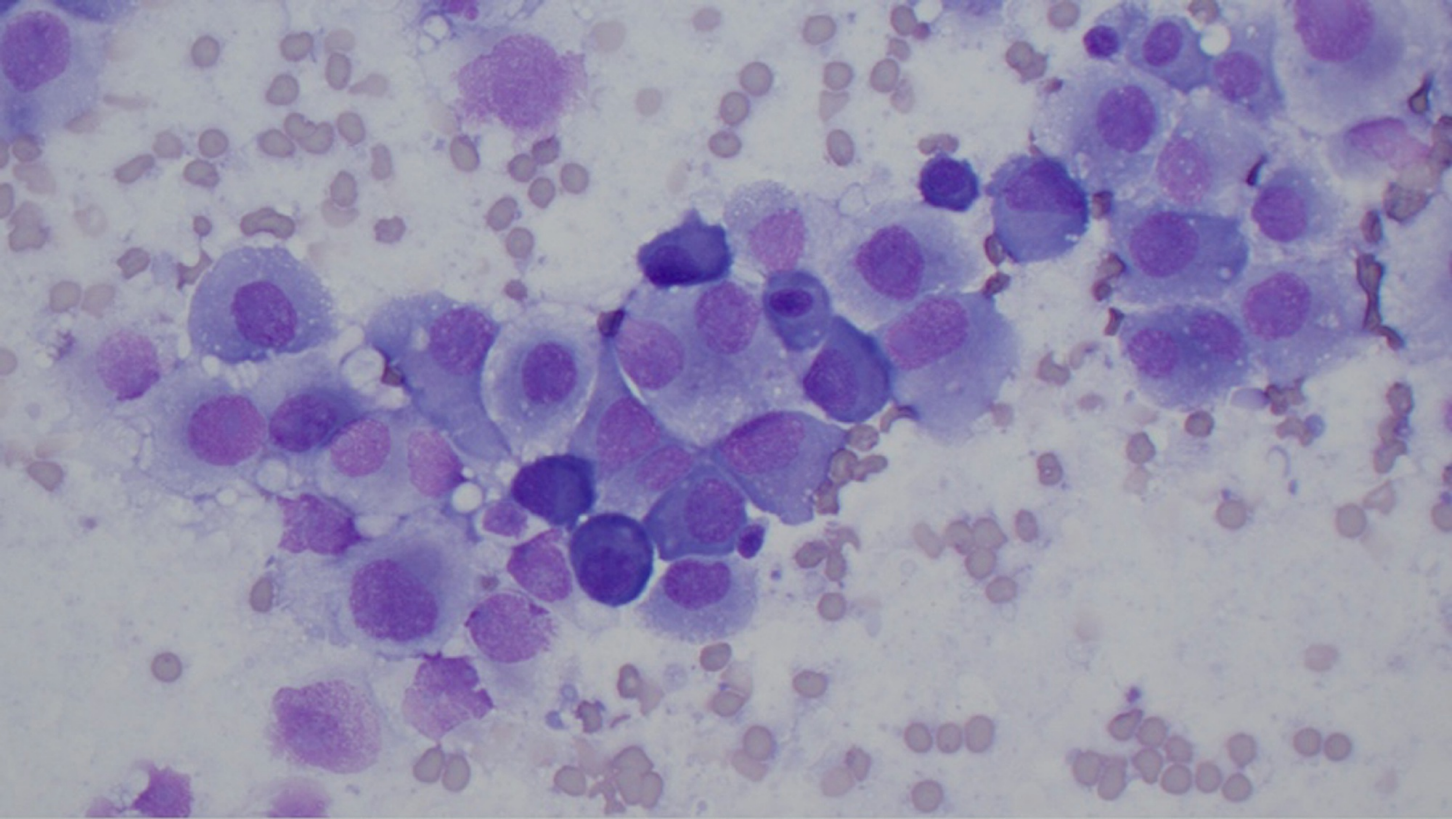
Intraoperative smear showing sheets of atypical plasma cells with eccentrically located nuclei, round nuclear contour, fine chromatin, prominent large nucleoli, and moderate amount of light blue cytoplasm. The binucleated form was also present (Diff-Quik stain, 400×).

Neurointerventional radiology was consulted, and a CT-guided biopsy of the lesion was scheduled to identify the nature of the mass, that is, squamous cell carcinoma metastasis versus meningioma versus plasmacytoma, as treatment of a plasmacytoma would involve radiation and medical therapy as opposed to a craniotomy for tumor resection. The patient was taken to the radiology suite and positioned supine on the CT table. General anesthesia was used for the duration of the procedure. A CT of the base of the skull was conducted with intravenous contrast to identify the right internal carotid and right ophthalmic arteries and their branches. The images demonstrated an expansive enhancing mass in the right cavernous sinus, extending into the right middle cranial fossa, right orbit, and right sphenoid sinus (Figure 3). The skin overlying the right maxillary sinus was prepped and draped using sterile protocol. Lidocaine (1%) with bicarbonate was used as a local anesthetic. A 17-gauge guiding needle was advanced through the right maxillary sinus, the right sphenoid sinus, and into the intracranial lesion (Figures 4, 5). The stylet was removed, and a 20-gauge biopsy needle was advanced through the guiding needle. Three biopsies were obtained and sent to pathology, which confirmed a diagnosis of plasmacytoma (Figures 6, 7). The patient was referred to radiation oncology that recommended 30 Gray (Gy) in 10 fractions to the intracranial lesion (1 Gy = 100 rads = 1 J/kg absorbed radiation).

## Discussion

Plasmacytomas occurring as extramedullary lesions in the cavernous sinus are quite rare. Cavernous sinus syndrome results from a mass effect within the cavernous sinus. Cranial nerves III (oculomotor), IV (trochlear), V1 (ophthalmic branch of trigeminal), V2 (maxillary branch of trigeminal), and VI (abducens) pass through the sinus in addition to the internal carotid artery and ocular sympathetic nerves [4,5]. Impingement of these neurovascular structures can cause orbital pain, diplopia, exophthalmos, and ptosis.

Typically, tumors of the cavernous sinus consist of meningiomas, pituitary adenomas, schwannomas, squamous cell carcinomas, and adenoid cystic carcinomas [6]. According to the current literature, plasmacytomas comprise 4% or less of tumors found in the cavernous sinus and often mimic metastasis [3,6]. These lesions are contrast-enhancing on MRI and may be associated with adjacent bony erosions. Because these tumors are uncommon, neurosurgeons tend to plan surgical resection without further workup of the lesion. Plasmacytomas are one of the few tumors that exhibit excellent response to radiotherapy and oftentimes do not require surgical debulking unless the lesion is compressing vital structures [7]. If properly identified, craniotomy with intracranial mass resection can be avoided.

Our case report is notable for two reasons. To our knowledge, fewer than 30 cases of plasmacytomas of the cavernous sinus have been reported in the literature [3]. Second, a neurointerventional biopsy via the maxillary bone and sinus allowed access to the center of the cavernous sinus lesion. To our knowledge, only one paper describes a transsphenoidal biopsy of a plasmacytoma [8]. Our procedure took approximately one hour using general anesthesia and a CT machine. Risks of this approach include bleeding, infection, as well as injury to the carotid artery and its branches if these are not identified intraoperatively [9]. Successful histological identification of a plasmacytoma (mature plasma cells) using a core biopsy changed the course of treatment from surgical to medical management, avoiding major neurosurgical resection [10]. We would like to note that an endoscopic transnasal biopsy was considered for this patient; however, the procedure would have been more invasive. Following CT biopsy, our patient had only a small mark on the skin and could proceed to therapy without any delay in recovery.

## Conclusions

While extramedullary plasmacytomas of the cavernous sinus are rare, we recommend a CT-guided biopsy to confirm or rule out the diagnosis in patients with confirmed or suspected multiple myeloma before proceeding with a craniotomy and mass resection. Management of a solitary plasmacytoma involves radiotherapy in combination with chemotherapy for the treatment of systemic multiple myeloma. Surgical resection of a solitary plasmacytoma is not recommended unless immediate debulking is indicated to decompress adjacent structures.
